# Adjuvant therapy with gemcitabine and stereotactic body radiation therapy versus gemcitabine alone for resected stage II pancreatic cancer: a prospective, randomized, open-label, single center trial

**DOI:** 10.1186/s12885-022-09974-7

**Published:** 2022-08-08

**Authors:** Tao Ma, Xueli Bai, Qichun Wei, Yongjie Shui, Mengyi Lao, Wen Chen, Bingfeng Huang, Risheng Que, Shunliang Gao, Yun Zhang, Wei Chen, Ji Wang, Tingbo Liang

**Affiliations:** 1 Department of Hepatobiliary and Pancreatic Surgery, Zhejiang Provincial Key Laboratory of Pancreatic Disease, the First Affiliated Hospital of Zhejiang University School of Medicine, 79 Qingchun Road, Hangzhou, 31003 China; 2 Department of Radiation Oncology, the Second Affiliated Hospital of Zhejiang University School of Medicine, Hangzhou, China

**Keywords:** Pancreatic adenocarcinoma, Adjuvant therapy, Gemcitabine, SBRT, Randomized controlled trial

## Abstract

**Background:**

The role of adjuvant radiation in pancreatic adenocarcinoma (PDAC) remains unclear. We aimed to investigate the efficacy of gemcitabine combined with stereotactic body radiation therapy (SBRT) as adjuvant therapy for resected stage II PDAC.

**Methods:**

In this single-center randomized controlled trial, patients with stage II PDAC that underwent margin-negative resection were randomly assigned to gemcitabine-alone adjuvant chemotherapy or adjuvant SBRT followed by gemcitabine chemotherapy. The primary endpoint was recurrence-free survival (RFS). Secondary endpoints included locoregional recurrence-free survival (LRFS), overall survival (OS), and incidence of adverse events.

**Results:**

Forty patients were randomly assigned to treatment between Sep 1, 2015 and Mar 31, 2018. Of these, 38 were included in the intention-to-treat analysis (20 in gemcitabine arm and 18 in gemcitabine plus SBRT arm). The median RFS and OS were 9.70, 28.0 months in the gemcitabine arm and 5.30, 15.0 months in the gemcitabine plus SBRT arm (RFS, *P* = 0.53; OS, *P* = 0.20), respectively. The median LRFS in both arms was unreached (*P* = 0.81). Grade 3 or 4 adverse events were all comparable between the two arms. Evaluation of data from the enrolled patients indicated that the addition of adjuvant SBRT was not associated with either better local disease control or recurrence-free survival.

**Conclusions:**

Adjuvant SBRT neither provided a survival benefit nor improved local disease control in resected stage II PDAC.

**Trial registration:**

ClinicalTrials.gov, NCT02461836. Registered 03/06/2015

## Introduction

Adjuvant chemotherapy has become the standard of care for all resected Pancreatic adenocarcinoma (PDAC) [1–3]. In contrast, the role of adjuvant radiotherapy for resected PDAC is still debatable. GITSG trial showed for the first time that adjuvant chemoradiation (CRT) significantly improved survival [4]. However, followed EORTC trial only showed a minor survival benefit of CRT [5]. ESPAC-1 trial even showed adjuvant CRT has a deleterious effect on survival [1]. Taken together, the benefit of adjuvant radiotherapy remains unclear.

Compared to traditional CRT, stereotactic body radiation therapy (SBRT) can preciously deliver a relatively high dose of radiation to the target tissue while minimizing radiation to surrounding tissue, in a hypofractionated manner [6]. SBRT was shown to have excellent local control and minimal toxicity while remaining cost-effective in treating PDAC [7]. It has been implemented as primary treatment in unresectable and recurrent PDAC, and as neoadjuvant treatment in locally advanced and borderline resectable disease [8–10]. However, the potential role of SBRT as adjuvant therapy for resected PDAC is still unknown.

Due to the shorter duration and excellent local control of SBRT treatment, it may become a promising adjuvant regimen for resected PDAC with less delay of systemic chemotherapy. Recently, we published our primary experience in adjuvant SBRT for resected PDAC, and the initial result was promising [11]. Here, we report the results from a prospective randomized single-center trial evaluating the potential benefit of adjuvant SBRT for resected stage II PDAC.

## Patients and methods

### Study design

This randomized controlled single-center trial (NCT02461836) was designed to compare the oncological benefit of adjuvant gemcitabine plus SBRT to gemcitabine alone for resected Stage II (AJCC TNM 7^th^) PDAC. The protocol was approved by the Medical Ethics Review Committee of our institution.

### Participants

Eligible patients were between 18 and 75 years of age with histologically proven stage II ductal pancreatic adenocarcinoma (according to the AJCC TNM Classification of Pancreatic Carcinoma, 7^th^ [12]); an Eastern Cooperative Oncology Group (ECOG) performance status [13] of 0 or 1; no history of neoadjuvant therapy; adequate bone marrow, hepatic, and renal function according to laboratory test results. Patients were excluded if they had undergone margin-positive resections or resection of recurrence PDAC; were subjected to severe postoperative complications; had serious co-morbidities; or if an investigator judged participation to be incompatible with the safety of the study.

### Randomization and masking

After confirming eligibility by the investigators, patients were randomized in a 1:1 ratio into the gemcitabine plus SBRT (GEM + SBRT) or the gemcitabine (GEM) arm, with a computer-generated random number list. The investigators were not masked to information regarding the allocation and treatment of patients. And the participants were aware of their group assignment. Data management and analysis were performed by independent analysts unrelated to this study.

### Treatment protocol

Patients assigned to the GEM arm received adjuvant gemcitabine chemotherapy while those assigned to the GEM + SBRT arm received SBRT before gemcitabine chemotherapy as the GEM arm did. Gemcitabine was delivered as a 1000 mg/m^2^ intravenous infusion administered once a week for three of every 4 weeks (one cycle) for six cycles (24 weeks). The radiation target field was delineated according to our previous method [11]. For the SBRT treatment plans, 25 Gy in 5-Gy fractions was delivered in the target area, ensuring more than 90% of each target volume received 100% of the prescription dose. SBRT was delivered at 4 ~ 10 weeks post-operatively after patient enrollment, and the time interval between the termination of SBRT and the initiation of gemcitabine chemotherapy was 1 ~ 2 weeks. Patients were assessed at 3-month intervals after enrollment for one year if alive at this point. The evaluation method of follow-up included lab tests of hematology, serum liver and renal function parameters, serum tumor markers, computed tomography, and magnetic resonance imaging.

### Endpoints

The primary endpoint was recurrence-free survival (RFS), measured as the time from operation until tumor recurrence or death. Patients without tumor recurrence and still alive at the point of final analysis were censored. Secondary endpoints included locoregional recurrence-free survival (LRFS) measured as the minimum time from operation to the date of local tumor recurrence, overall survival (OS), and incidence of adverse events (AEs). Locoregional recurrence was defined as recurrence in the pancreatic remnant, pancreatic anastomosis, local connective tissue, and regional lymph nodes.

### Sample size calculation

On the basis of previously published data from the CONKO-001 trial [2], we assumed that the median RFS would be 11 months for the GEM arm, and 17 months for the GEM + SBRT arm. We calculated that the total sample size needed to be 512 eligible patients based on 80% power with 5% two-sided α risk. The sample size was inflated to account for patient withdrawals and lost to follow-up (10%) at the time of analysis.

### Statistical analysis

All data were analyzed using SPSS 21.0 (SPSS, Chicago, IL). The χ2 and Fisher’s exact probability tests were used to analyze the differences between qualitative data, and the Mann–Whitney U test for the differences between quantitative data. Survival rates were calculated using the Kaplan–Meier method, and the log-rank test was used to analyze the differences. A *P*-value less than 0.05 was considered statistically significant.

## Results

### Patient characteristics

**Fig. 1:**
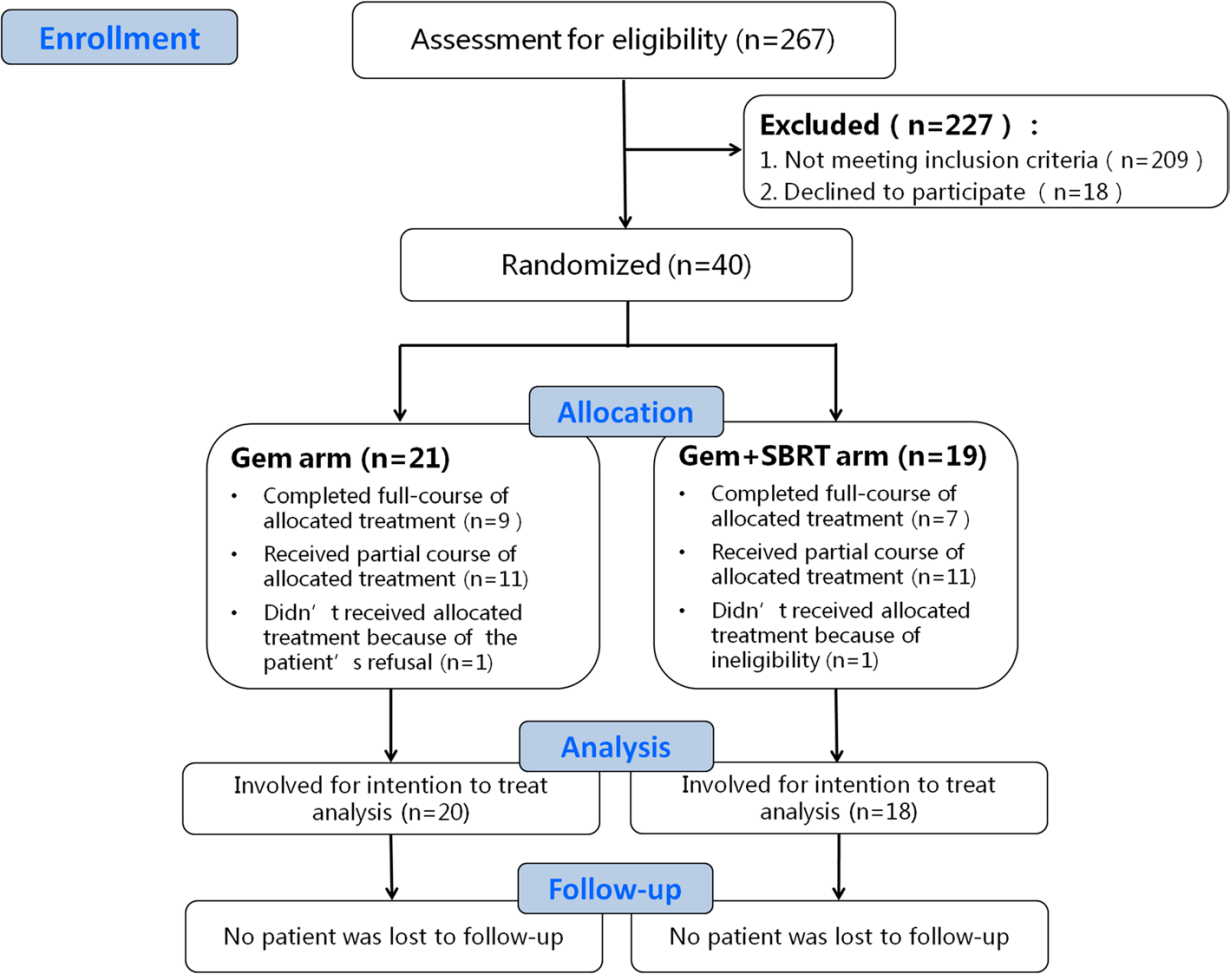
The CONSORT flow diagram for this trial

Forty patients were enrolled from Sep 1, 2015 to Mar 31, 2018. Of these, 38 were included in the intention-to-treat analysis (20 in the GEM arm and 18 in the GEM + SBRT arm) (Fig. 1). Both arms were well balanced regarding baseline characteristics such as age, sex, general status, and tumor size. The median follow-up time was 31.0 (IQR 21.0 – 43.2) months for the GEM arm and 25.0 (IQR 21.0–34.8) months for the GEM + SBRT arm. The median time from surgery to chemotherapy is 41.5 (IQR 35.5 – 45.0) days in the GEM arm, and 62.5 (IQR 51.0 – 69.3) days in the GEM + SBRT arm (*P* < 0.001) (Table 1).

**Table 1:** Patient demographics and clinical characteristics

	**GEM arm** **(*n* = 20)**	**GEM + SBRT arm** **(*n* = 18)**	***P***
Age, y, median (IQR)	58.5 (50.3–67.3)	63.0 (58.8–66.0)	0.176
Sex, male, n (%)	12 (60.0%)	13 (72.2%)	
BMI, Kg/m^2^, median (IQR)	22.2 (20.4–25.0)	23.3 (22.0–24.8)	0.361
ECOG, n (%)			0.825
0	14 (70.0%)	12 (66.7%)	
1	6 (30.0%)	6 (33.3%)	
Preoperative CA19-9, U/L, median (IQR)	267.9 (38.8–737.3)	575.3 (105.8–1921.0)	0.305
CA19-9 on randomization, U/L, median (IQR)	24.4 (5.3–105.5)	126 (7.2–375.5)	0.160
Post-operative complications (Clavien-Dido ≥ grade III), n (%)	5 (25.0%)	7 (38.9%)	0.358
Surgery to adjuvant chemotherapy, d, median (IQR)	41.5 (35.5 – 45.0)	62.5 (51.0 – 69.3)	< 0.001
Number of dissected lymph nodes, median (IQR)	20.0 (12.3 – 23.8)	20.5 (16.5 – 29.3)	0.276
Number of positive lymph nodes, median (IQR)	1.00 (0–3.0)	2.00 (0.8 – 4.0)	0.534
Number of node-positive patients, n (%)	12 (60.0%)	13 (72.2%)	0.428
Maximum tumor size, mm, median (IQR)	3 (2.3–3.9)	3 (2.4–3.7)	0.828
Procedure, n (%)			0.084
Pancreaticoduodenectomy	13 (65.0%)	16 (88.9%)	
Distal pancreatectomy	7 (35.0%)	2 (11.1%)	
Combined vascular resection, n (%)	5 (25.0%)	6 (33.3%)	0.572
Combined organ resection, n (%)	1 (5.0%)	0	0.336

*BMI* Body-mass index, *ECOG* Eastern Cooperative Oncology Group, *GEM* Gemcitabine, *IQR* Interquartile range, *SBRT*, stereotactic body radiation therapy

### Primary endpoint

**Fig. 2:**
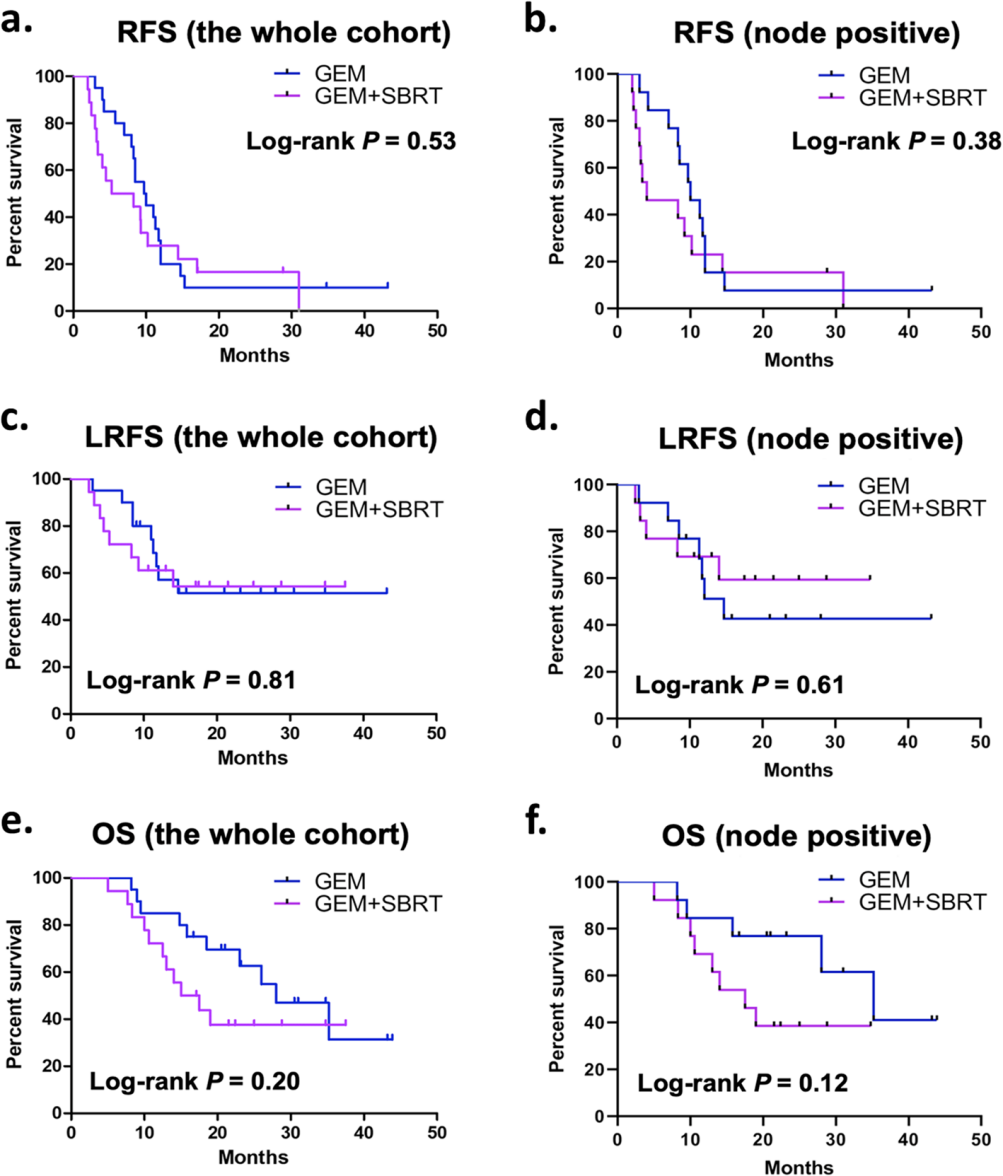
Recurrence-free survival (RFS) (**a**), locoregional recurrence-free survival (LRFS) (**c**), and overall survival (OS) (**e**) of the whole cohort; and RFS (**b**), LRFS (**d**), and OS (**f**) of node-positive patients

Out of the 38 patients who were involved in the intention-to-treat analysis, 18 (18/20) in the GEM arm and 16 (16/18) in the GEM + SBRT arm had tumor recurrence before the last follow-up. The incidences of different sites of recurrence were all comparable between the two arms (Table 2). The median RFS was 9.70 (IQR 7.00–12.00) months in the GEM arm and 5.30 (IQR 3.20–14.40) months in the GEM + SBRT arm. The Hazard ratio (HR) for tumor recurrence of GEM, compared with GEM + SBRT, was 0.80 (95% CI 0.40–1.60, *P* = 0.53) (Fig. 2-a). In patients with node-positive PDAC (*n* = 26), the median RFS was 10.0 (IQR 8.30—12.0) months in the GEM arm (*n* = 13) and 4.0 (IQR 3.0 – 10.2) in the GEM + SBRT arm (*n* = 13), and the HR for tumor recurrence of GEM, compared with GEM + SBRT, was 0.71 (95% CI 0.31 – 1.59, *P* = 0.38) (Fig. 2-b).

**Table 2:** Recurrence pattern

	**Whole Cohort (*n* = 38)**	**GEM Arm** **(*n* = 20)**	**GEM + SBRT Arm (*n* = 18)**	***P***
Number of patients with recurrence, n (%)	34 (89.5%)	18 (90.0%)	16 (88.9%)	0.911
All recurrence events, n	42	22	20	-
Locoregional recurrence, n (%)	15 (35.7%)	9 (40.9%)	6 (30.0%)	0.463
Systemic recurrence, n (%)				
Liver	15 (35.7%)	6 (27.3%)	9 (45.0%)	0.208
Lung	4 (9.5%)	3 (13.6%)	1 (5.0%)	0.344
Peri-aortic lymph node	3 (7.1%)	1 (4.6%)	2 (10.0%)	0.485
Peritoneum	5 (11.9%)	3 (13.6%)	2 (10.0%)	0.723

### Secondary endpoints

The median LRFS in both of the two arms was unreached. The HR for local recurrence of GEM, compared with GEM + SBRT, was 0.89 (95% CI 0.34 – 2.32, *P* = 0.81) (Fig. 2-c). The median OS was 28.0 (95% CI 18.18–37.82) months in the GEM arm and 15.0 (95% CI 10.16–19.84) months in the GEM + SBRT arm. The HR for death of GEM, compared with GEM + SBRT, was 0.56 (95% CI 0.23 – 1.36, *P* = 0.20) (Fig. 2-e). In patients with node-positive PDAC (*n* = 26), the median LRFS was 14.7 (95% CI 9.78 – 19.62) months in the GEM arm (*n* = 13) and unreached in the GEM + SBRT arm (*n* = 13), and the HR for local recurrence of GEM, compared with GEM + SBRT, was 1.35 (95% CI 0.43 – 4.17, *P* = 0.61) (Fig. 2-d). Also, in patients with node-positive PDAC (*n* = 26), the median OS was 35.2 (IQR 21.38 – 49.02) months in the GEM arm (*n* = 13) and 17.5 (IQR 10.45 – 24.55) in the GEM + SBRT arm (*n* = 13), and the HR for death of GEM, compared with GEM + SBRT, was 0.43 (95% CI 0.14 – 1.31, *P* = 0.12) (Fig. 2-f). Grade 3 or 4 AEs observed in the whole cohort were comparable between the two groups (Table 3).

**Table 3:** Adverse events (AE) (≥ grade III)

AE (≥ grade III), n	Whole cohort (*n* = 38)	GEM arm (*n* = 20)	GEM + SBRT arm (*n* = 18)	*P*
Neutropenia	3	2	1	0.612
Thrombocytopenia	1	0	1	0.285
Fatigue	1	0	1	0.285
Anorexia	3	1	2	0.485
Anaemia	0	0	0	-
Nausea or vomitting	5	3	2	0.723
Diarrhea	0	0	0	-
Liver dysfunction	3	2	1	0.612

Evaluation of data from the enrolled patients indicated that the addition of adjuvant SBRT was not associated with either better local disease control or recurrence free survival. As a result, discontinuation of this study was determined.

## Discussion

To our knowledge, this is the first randomized controlled trial to evaluate the potential benefit of SBRT for PDAC in the adjuvant setting. The role of adjuvant radiotherapy in PDAC has long been a debatable issue, and the survival benefit of adding radiation to gemcitabine in the adjuvant setting has not been conclusively shown. Even different guidelines have different recommendations. The American Society of Clinical Oncology (ASCO) guidelines recommend the addition of adjuvant CRT to systemic chemotherapy for patients with node-positive or margin-positive disease [14]. In contrast, the European Society of Medical Oncology (ESMO) guidelines don’t recommend adjuvant CRT, except in clinical trials [15].

Although until now, there’s no convincing data of the survival benefit of adjuvant radiation in PDAC. Emerging new evidence is indicating that adjuvant CRT may benefit pancreatic patients. The ORTC-FFCD-GERCOR trial suggests that gemcitabine-based CRT is feasible, well-tolerated, and not deleterious. Adding CRT into adjuvant chemotherapy results in less frequent first local recurrence and simultaneous local and distant progression. There are no significant differences in RFS or OS [16]. Recently, Kamarajah et al. found adjuvant radiation was associated with better survival. And stratified and multivariable interaction analyses showed that this benefit was restricted to node-positive disease [17]. In a recent retrospective study, patients receiving increasing doses of adjuvant CRT after surgical resection with macroscopically negative margins showed a significantly improved OS [18]. However, in the present study, adjuvant SBRT failed to show an advantage over gemcitabine alone in terms of local tumor control and survival in stage II pancreatic patients. Even in a subgroup analysis for node-positive patients, there is also no apparent benefit of adjuvant SBRT. These findings may be partially explained by different radiotherapy modalities and dosages, treatment protocols, and patient population. And the relatively low efficacy of gemcitabine monotherapy may also contribute to the failure of the combined therapy, as combination regimens (such as gemcitabine and capecitabine, FOLFIRINOX, etc.) have been proven to be more efficient in the adjuvant setting.

Efficient local control is of importance to prevent regional recurrence and minimize risks of distant failure [19, 20]. The effort of local control may be hampered by a delayed application of radiotherapy after surgery, especially in node-positive, outside growth, or margin-positive tumors. In such circumstances, the risk of early tumor relapse is much more common, and as a result, the application of additional radiation is usually given way to stronger systemic therapies for recurrence tumors. Unlike traditional modalities of radiotherapy, the major advantage of SBRT is its capacity of delivering a high biologically effective dose while minimizing the dose to surrounding tissue in a few days [6]. These characteristics make SBRT a good choice for rapid delivery of radiation prior to systemic chemotherapy while omitting the risk of interrupting the initiation of chemotherapy. In this study, the total dose of SBRT was 25 Gy in 5 fractions. It was optimistically estimated to be finished in 5 days to minimize interruption to chemotherapy. However, the interruption was markedly underestimated, as shown in this study the median delay of chemotherapy was 21 days. Another concern is the relatively low dose of SBRT. A recent study demonstrates increasing doses of CRT is associated with better survival [18]. Actually, in some studies showing improved survival with the use of adjuvant CRT, the cumulative doses were 50 Gy or even higher [21, 22]. While in EORTC and ESPAC-1 trials that showed no or even deleterious effect of CRT, the cumulative dose was both 40 Gy [5, 23]. Taken together, the underestimated interruption to chemotherapy and a relatively low dose of SBRT might contribute to the failure of this trial.

In this trial, the incidence of first distal metastasis was much more common than first locoregional recurrence. Although it’s well recognized that locoregional recurrence is difficult to get detected unequivocally on imaging, the large proportion of distal metastasis indicated the systemic nature of tumor relapse in the trial population, which may also add to the explanation of SBRT’s failure. Considering neoadjuvant chemotherapy and radiation become more commonly used, resulting in higher rates of systemic control and nodal sterilization prior to surgery, the benefit of local control in the adjuvant setting may become even less attractive.

This study has several limitations that should be acknowledged. First, it’s a single-center trial, which may potentially limit external validity. Another limitation is related to the small sample size, which impaired a robust analysis. Finally, the study design may hamper the ability to get the predicted results, including the relatively lower dose of SBRT and the unexpected longer delay of initiation of chemotherapy.

## Conclusions

This single-center, randomized controlled clinical trial showed that the addition of SBRT in the adjuvant setting didn’t improved local control of disease or patients’ survival in stage II PDAC.

## Data Availability

The datasets generated and/or analysed during the current study are not publicly available due to privacy/ethical restrictions. but are available from the corresponding author on reasonable request.

## Abbreviations

AEs: Adverse events
CRT: Chemoradiation
ECOG: Eastern Cooperative Oncology Group
GEM: Gemcitabine
LRFS: Locoregional recurrence-free survival
OS: Overall survival
PDAC: Pancreatic adenocarcinoma
RFS: Recurrence-free survival
SBRT: Stereotactic body radiation therapy
